# Pseudoaneurysm of the Internal Pudendal Artery Causing Delayed Vaginal Hemorrhage After Robotic-Assisted Total Hysterectomy: A Case Report

**DOI:** 10.7759/cureus.108929

**Published:** 2026-05-15

**Authors:** Tomoko Ueda, Tomoyuki Sasano, Hiroshi Kodama, Sachiyo Narita, Hiroshi Tsubamoto

**Affiliations:** 1 Obstetrics and Gynecology, Hyogo Medical University School of Medicine, Nishinomiya, JPN; 2 Radiology, Hyogo Medical University School of Medicine, Nishinomiya, JPN; 3 Gynecology, Takarazuka City Hospital, Takarazuka, JPN

**Keywords:** delayed hemorrhage, internal pudendal artery, pseudoaneurysm, robotic hysterectomy, vaginal laceration

## Abstract

Pseudoaneurysm is a rare but potentially life-threatening cause of delayed postoperative hemorrhage. Although pseudoaneurysms are well described after obstetric and gynecologic procedures, involvement of the internal pudendal artery after hysterectomy is extremely rare. A 50-year-old nulligravid woman underwent robot-assisted total hysterectomy with bilateral salpingectomy for symptomatic uterine leiomyomas. During transvaginal specimen retrieval, vaginal wall lacerations occurred bilaterally and were sutured intraoperatively. The postoperative course was uneventful, and the patient was discharged on postoperative day 5. On postoperative day 22, she presented with vaginal bleeding originating from the sutured site on the right vaginal wall. Initial transvaginal suturing achieved temporary hemostasis; however, recurrent bleeding occurred later the same day. Three-dimensional computed tomography angiography revealed active extravasation from a pseudoaneurysm arising from a branch of the right internal pudendal artery. Transcatheter arterial embolization using gelatin sponge particles successfully controlled the bleeding. Transvaginal specimen retrieval caused pseudoaneurysm formation of the internal pudendal artery. Transcatheter arterial embolization is an effective and minimally invasive treatment option.

## Introduction

A pseudoaneurysm is a vascular lesion that occurs when an arterial wall is disrupted, allowing blood to leak into the surrounding tissue and form a pulsatile hematoma encapsulated by fibrous tissue rather than a true arterial wall. In obstetrics and gynecology, pseudoaneurysms are most commonly reported in the context of postpartum hemorrhage and typically involve the uterine artery [1]. Reports of pseudoaneurysms arising from the internal pudendal artery are rare [2]. Pseudoaneurysm formation after hysterectomy has also been described, although it remains uncommon, with most reported cases involving the uterine artery [3-5]. Here, we report a rare case of delayed vaginal hemorrhage caused by a pseudoaneurysm of the internal pudendal artery following robotic-assisted total hysterectomy.

## Case presentation

A 50-year-old nulligravid woman (height, 159 cm; weight, 53 kg; body mass index (BMI), 21 kg/m²) was referred to our hospital for evaluation of menorrhagia and anemia. During menstruation, she required changing overnight sanitary pads every hour and had been receiving periodic intravenous iron therapy for anemia. Pelvic examination revealed an enlarged uterus approximately the size of a clenched fist. Magnetic resonance imaging (MRI) demonstrated multiple intramural and submucosal leiomyomas. The uterine dimensions on preoperative MRI were 5.5 × 10.2 × 6.8 cm.

**Figure 1:**
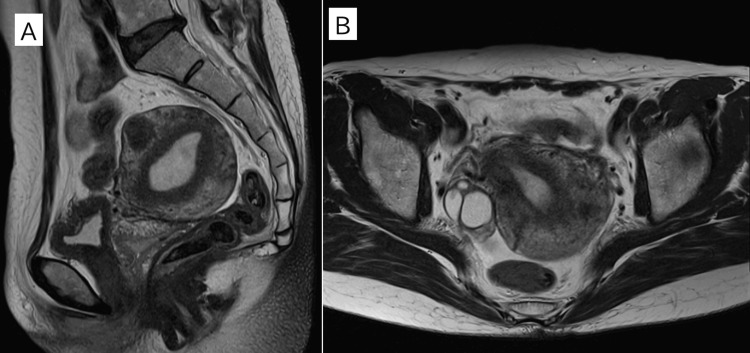
Preoperative magnetic resonance imaging findings. (A) Sagittal T2-weighted magnetic resonance image demonstrating an enlarged uterus with multiple intramural leiomyomas showing heterogeneous low-to-intermediate signal intensity. (B) Axial T2-weighted magnetic resonance image showing multiple uterine leiomyomas and a mildly thickened endometrium. MRI: magnetic resonance imaging

The endometrium was mildly thickened; however, an outpatient endometrial biopsy using a suction pipette showed no evidence of hyperplasia or malignancy (Figure 1).

The patient requested definitive surgical management and underwent robot-assisted total hysterectomy with bilateral salpingectomy using the da Vinci Xi system. During transvaginal specimen retrieval, resistance was encountered, likely due to her nulligravid status; however, the uterus was successfully removed without morcellation. This maneuver resulted in bilateral vaginal wall lacerations. These lacerations were sutured transvaginally; the laceration on the left side was deeper and required additional time to achieve hemostasis. Adequate hemostasis was ultimately confirmed. The total operative time was two hours and 30 minutes, with minimal blood loss. Postoperatively, no vaginal bleeding was observed, and the postoperative course was uneventful. The patient was discharged on postoperative day 5.

**Figure 2:**
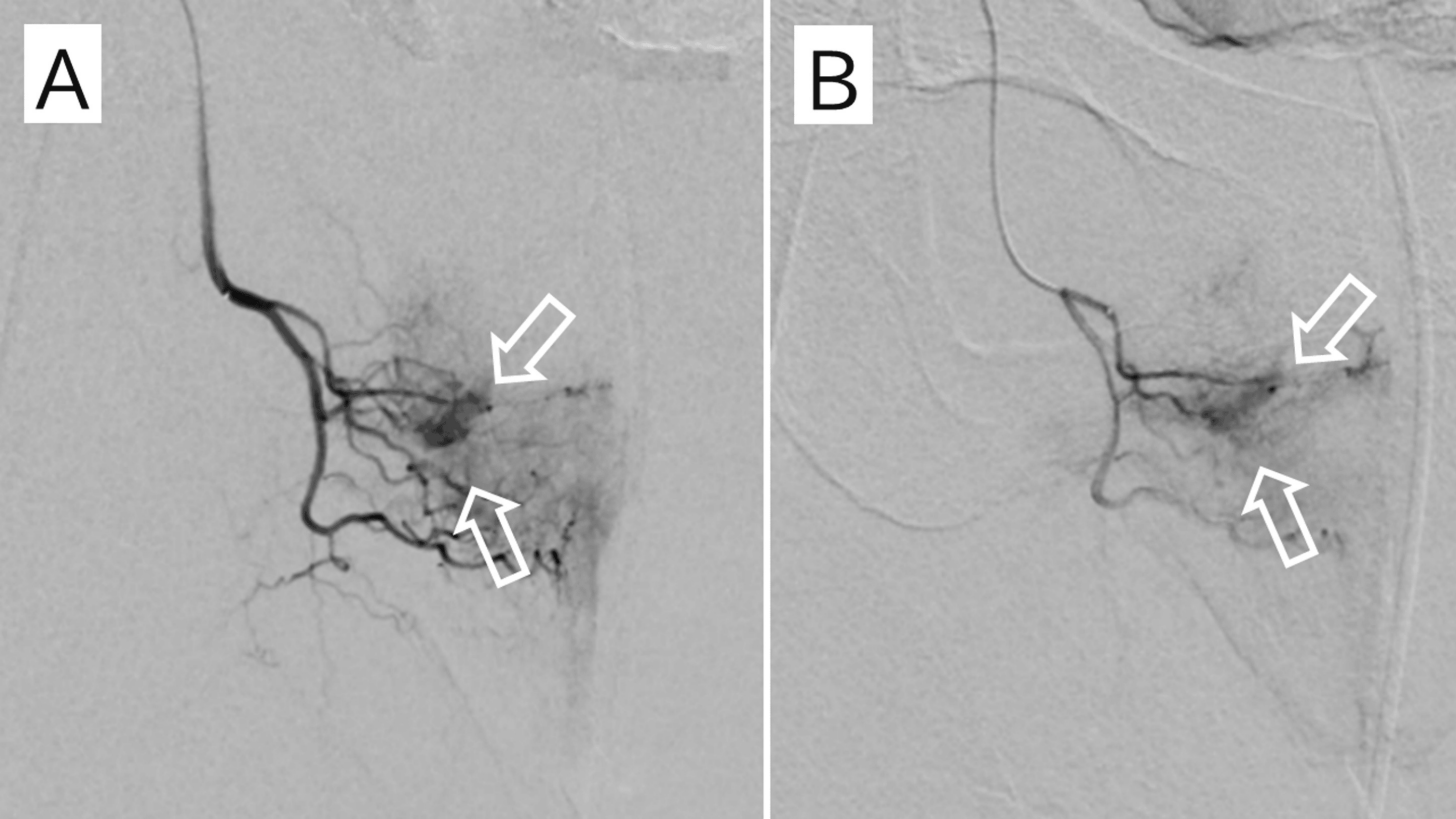
Angiographic findings of pseudoaneurysm and embolization. (A) Selective angiography demonstrates active contrast extravasation from a branch of the right internal pudendal artery (arrows), consistent with a pseudoaneurysm. (B) After transcatheter arterial embolization using gelatin sponge particles, complete cessation of contrast extravasation is observed (arrows). Bilateral internal iliac artery angiography confirmed no additional bleeding sources.

On postoperative day 22, the patient presented with vaginal bleeding from the right vaginal wall. The exact source of rebleeding was unclear at that time, and the vaginal lacerations were repaired using 0 Vicryl® sutures (Ethicon; Raritan, NJ, USA) with both interrupted and continuous techniques. She was discharged; however, recurrent bleeding occurred after two hours, prompting readmission. Multiphasic contrast-enhanced computed tomography revealed active extravasation along the right vaginal wall. Angiography was performed via a femoral approach using a 4-Fr catheter and a 1.7-Fr microcatheter (Progreat λ; Terumo Corp., Tokyo, Japan). Selective angiography of a branch of the right internal pudendal artery demonstrated active extravasation, and the vessel was embolized using gelatin sponge particles (Serescue; Nippon Kayaku Co., Ltd., Tokyo, Japan), resulting in complete cessation of bleeding (Figure 2).

**Figure 3:**
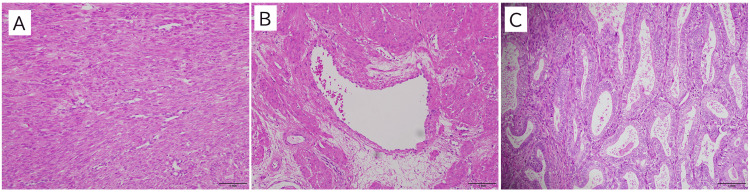
Histopathological findings of the surgical specimen. (A) Leiomyoma composed of interlacing bundles of uniform spindle-shaped smooth muscle cells without significant atypia (hematoxylin and eosin (H&E) staining, ×200). (B) Adenomyosis characterized by the presence of endometrial glands and stroma within the myometrium (H&E staining, ×200). (C) Incidental grade 1 endometrioid carcinoma confined to the endometrium without myometrial invasion or lymphovascular invasion (H&E staining, ×200). Scale bar = 100 μm.

Histopathological examination of the surgical specimen revealed leiomyomas and adenomyosis, as well as an incidental 7-mm International Federation of Gynecology and Obstetrics (FIGO) stage IA grade 1 endometrioid carcinoma without myometrial invasion or lymphovascular invasion (Figure 3).

After discussion with the patient, an additional laparoscopic bilateral salpingo-oophorectomy was performed. No residual malignancy was identified on pathological examination. During two years of follow-up, the patient has remained without recurrent hemorrhage or vascular complications and has remained disease-free, with no evidence of cancer recurrence.

## Discussion

Pseudoaneurysm is an uncommon but clinically significant cause of delayed hemorrhage in gynecologic practice. It results from arterial wall injury followed by the formation of a contained hematoma that maintains communication with the parent vessel, and rupture may occur days to weeks after the initial insult. In gynecology, most reported pseudoaneurysms involve the uterine artery and vaginal artery [3,6]. Delayed hemorrhage due to uterine artery pseudoaneurysm has been described after various procedures, including open or laparoscopic hysterectomy, hysteroscopic surgery, myomectomy, and dilation and curettage [7-9].

In contrast, pseudoaneurysms originating from the internal pudendal artery are exceedingly rare. The internal pudendal artery supplies the perineum and vaginal wall, and injury may occur during vaginal delivery, vaginal manipulation, suturing, or traction [2]. To our knowledge, no previous reports have described pseudoaneurysm of the internal pudendal artery following robotic-assisted or laparoscopic hysterectomy.

In the present case, resistance during transvaginal specimen extraction resulted in vaginal wall lacerations, with a deeper injury on the left side. However, the pseudoaneurysm developed on the right side, suggesting that the site of vascular injury may not always correspond to the more apparent or severe vaginal damage. It is possible that traction or manipulation during specimen retrieval caused subclinical injury to a branch of the internal pudendal artery on the contralateral side. In addition, suturing of the vaginal wall lacerations may have contributed to vascular injury in adjacent or deeper tissues not directly visible intraoperatively.

Diagnosis of a pseudoaneurysm can be challenging because the clinical presentation is often nonspecific. As highlighted in previous reports, a pseudoaneurysm may mimic other postoperative complications, and delayed vaginal bleeding may be the only presenting symptom. Early recognition is essential, as pseudoaneurysms may lead to life-threatening hemorrhages. Therefore, pseudoaneurysm rupture should be considered in the differential diagnosis when delayed or recurrent bleeding occurs after gynecologic surgery, particularly following transvaginal manipulation. Imaging modalities, including contrast-enhanced computed tomography and angiography, are essential for accurate localization of the bleeding source [10].

In the present case, it is likely that a pseudoaneurysm had already formed at the time of the initial bleeding episode on postoperative day 22; however, it was not recognized immediately, and transvaginal suturing was performed. Although a massive hemorrhage did not occur, the potential risk could not be excluded. In cases of uterine artery pseudoaneurysm, Doppler ultrasonography has been reported to be useful for diagnosis by demonstrating characteristic turbulent flow within the lesion [6]. However, in clinical practice, the bleeding source is often unclear, particularly in cases of deep pelvic hemorrhage. In such situations, contrast-enhanced computed tomography and angiography are the most valuable modalities for accurate localization and treatment planning.

In this case, the bleeding originated not from the vaginal cuff but from the sutured site of a right vaginal wall laceration. Although a pseudoaneurysm of the internal pudendal artery had not been initially considered in the differential diagnosis, the bleeding site was approximately predictable, and contrast-enhanced CT was performed with the intention of subsequent transcatheter arterial embolization. Prompt multidisciplinary collaboration is essential for optimal management.

The present case also highlights the importance of careful intraoperative decision-making during transvaginal specimen retrieval. In nulligravid patients or in cases where resistance is encountered during vaginal extraction, excessive traction, blind manipulation, and deep suturing within a narrow vaginal canal should be avoided whenever possible because these maneuvers may cause not only vaginal lacerations but also unrecognized vascular injury and subsequent pseudoaneurysm formation.

At the same time, this case also demonstrated incidental endometrioid carcinoma despite negative preoperative endometrial biopsy findings. Therefore, even in cases preoperatively considered benign, the possibility of occult focal malignancy should be recognized. From this perspective, when transvaginal extraction is difficult or resistance is encountered, contained in-bag morcellation may represent a reasonable alternative approach to reduce traction-related injury while minimizing the potential risk of intraperitoneal tissue dissemination.

## Conclusions

We report a rare case of delayed vaginal hemorrhage caused by a pseudoaneurysm of the internal pudendal artery following robot-assisted hysterectomy. This case highlights that excessive traction, blind manipulation, and deep suturing during difficult transvaginal specimen retrieval may result in unrecognized vascular injury and subsequent pseudoaneurysm formation. Therefore, a pseudoaneurysm should be considered in the differential diagnosis when delayed or recurrent bleeding occurs after transvaginal manipulation. In cases where vaginal extraction is difficult, alternative approaches, such as contained in-bag morcellation, may help reduce traction-related injury while minimizing potential oncologic risk. Prompt contrast-enhanced imaging and transcatheter arterial embolization provide effective and minimally invasive management for definitive hemostasis.
